# Utilizing large language models and natural language processing to classify ischemia status from cardiac stress tests in a large multicenter healthcare system

**DOI:** 10.1186/s13104-025-07586-5

**Published:** 2025-12-03

**Authors:** Shayna Cave, Kelly S. Peterson, Mary E. Plomondon, Stephen W. Waldo

**Affiliations:** 1 CART Program, Office of Quality and Patient Safety, Veterans Health Administration, Washington, DC USA; 2 Rocky Mountain Regional Veterans Affairs Medical Center, 1700 N Wheeling St, Suite F2-166, Aurora, CO 80045 USA; 3 Division of Cardiology, Department of Medicine, University of Colorado, Aurora, CO USA; 4 Department of Internal Medicine, Division of Epidemiology, University of Utah, Salt Lake City, UT USA

**Keywords:** Ischemia, NLP, LLM, Text classification, Rules-based, Cardiovascular

## Abstract

**Objective:**

Documentation of myocardial ischemia prior to invasive coronary angiography is recommended to minimize patient risk. However, obtaining this information for quality-of-care assessment often requires extracting clinical information from unstructured electronic medical records text. To this end, we sought to evaluate multiple natural language processing (NLP) systems in their ability to classify cardiac stress test reports as documenting ischemia or no ischemia, implementing the one with the best combination of accuracy and feasibility.

**Results:**

Four BERT large language models (LLMs) were fine-tuned, and a rules-based system was designed by training, validating, and testing on an annotated sample of 654 stress test reports from a multisite and multiyear dataset from the Veterans Health Administration (VHA). The LLM with the highest performance was a ClinicalBERT with precision, recall, and F1 of 86.4%, 100%, and 92.7%, respectively. The rules-based NLP system achieved similar results of 88.1%, 97.4%, and 92.5%, respectively. Stress test reports totaling 1,692,171 and representing 1,096,341 unique patients were classified using the rules-based system after ascertaining current technological limitations, and the system is presently operational for care quality evaluations. Utilizing NLP allows for accurate, high-throughput analysis of cardiac stress test text reports.

**Supplementary Information:**

The online version contains supplementary material available at 10.1186/s13104-025-07586-5.

## Introduction

Reduced blood flow to the heart muscle results in myocardial ischemia, which can clinically manifest as chest pain, shortness of breath, or as a myocardial infarction, otherwise known as a heart attack. While coronary angiography serves as the gold standard to evaluate the presence of ischemia, directly visualizing flow through the coronary arteries that supply blood to the myocardium, it is invasive and increases patient risk. Therefore, professional societies have adopted appropriate-use criteria to determine when this test may be warranted [1–4]. Based on these recommendations, invasive coronary angiography is only appropriate for patients with stable disease who already have non-invasive evidence of myocardial ischemia – most commonly, evidence from a cardiac stress test. Unfortunately, the medical record does not document the presence or absence of myocardial ischemia detected on non-invasive testing in a discrete data field that is readily available to monitor the appropriateness of invasive cardiovascular procedures on a population level.

One of the most common types of cardiac stress testing visualizes the distribution of a nuclear radiotracer throughout the myocardium at rest and during real or simulated levels of exertion. Resulting documentation of myocardial ischemia is heterogeneous and predominantly stored as free text. However, automated systems such as natural language processing (NLP) can be used to quickly and accurately extract clinical results from within report texts [5, 6], reducing time-consuming manual chart reviews.

To the best of our knowledge, only one other group has reported on a rules-based NLP system for specifically identifying ischemia from cardiac stress test reports [7–9], and no literature exists on doing so using large language models (LLMs). Previous NLP extraction of cardiac diagnostic information, sometimes including ischemia [10], from a variety of other text types such as outpatient notes [10, 11], hospital discharge notes [11], and cardiac magnetic resonance reports [12, 13] exhibit how valuable the approach is.

Creating an automated text processing system within the Veterans Health Administration (VHA) presents a variety of challenges. The VHA is the largest integrated health care system in the United States, providing care at 1380 health care facilities, including 170 VA Medical Centers and 1193 outpatient sites of care of varying complexity to over 9.1 million Veterans enrolled in the VA health care program, all with different patterns of documentation. Any NLP system must be able to address both the scale and complexity of this healthcare system with the governmental resources available. Here we evaluate two such solutions to the identification of ischemia in cardiac stress tests.

## Methods

### Data

VHA’s Corporate Data Warehouse (CDW) was used to obtain report texts and patient information. We identified users of VHA who had received a cardiac stress test report between October 1st, 2010 and September 21st, 2023. A cardiologist reviewed all procedure names to identify cardiac stress tests; both exercise and chemically induced stress tests were included as long as nuclear imaging data was available. Stress test report text data was obtained from two fields: impression text and full report text, with impressions having notably shorter and more succinct conclusions than the full reports. Both texts were obtained, but only the impressions were trained on and run in the NLP systems, as these were found to be less prone to false positives.

The analysis was performed in an operational capacity for the Department of Veterans Affairs, and thus institutional review board approval and informed consent was deemed unnecessary by the local authorities.

### Annotations

Annotations were completed in two rounds. The first set of annotations was a simple random sample of cardiac stress test reports selected from 10/1/2010 to 5/31/2023. The second round of annotations consisted of a stratified random sample from 10/1/2010 to 9/21/2023 from an updated dataset, ensuring no overlapping data points and inclusion of all available medical centers that perform cardiac stress tests. This was done to ensure generalizability to all VA sites performing stress tests, as linguistic distributions vary by facility [14–18], time period, and ischemia status. The second round of annotation data was additionally enriched for reports suspected of being positive for ischemia based on string searches, to better encapsulate the range of terms or phrases that could be used to designate a test as positive for ischemia. Oversampling the minority and undersampling the majority are common and long-standing methods used to combat imbalanced categories, improving model performance [19–21]. The first dataset was used as the training set, and the second dataset was randomly broken into the validation and test set.

Annotations were performed in these same two rounds by a practicing interventional cardiologist (SWW). Documents were categorized as positive or negative for ischemia. The statistician NLP developer reviewed all annotations for general accuracy and conferred with the physician to adjust any potential errors.

### NLP systems

Using the HuggingFace infrastructure, the Transformers Python packages [22], an 8GB GPU, and the training dataset, we fine-tuned four pre-trained transformer models in the BERT family [23] including DistilBERT Base Uncased and Cased [24], Bio + Clinical BERT [25], and ClinicalBERT [26]. The validation set was used for tuning hyperparameters such as learning rate, number of epochs, and weight decay. All evaluated models truncated the documents to 512 tokens. Breaking the texts up into multiple documents by token count was attempted but resulted in worse metrics. Clinical Longformer was tested but required greater computational capabilities to fine-tune than were available to us at the time.

The resulting LLMs produced a binary ischemia indicator for each stress test report. To evaluate performance, we used precision, recall, and F1 at the binary classification level. These metrics were compared among the LLMs as well as to a rules-based NLP pipeline.

The rules-based NLP system was constructed using the Python package medspaCy [27]. MedspaCys’ components and mechanisms have been documented in previous work [28], and our work builds on this. Rules were written in conjunction with specialty expertise from a practicing interventional cardiologist, requiring subject-specific rules of 33 target, 47 context, and 50 section rules as well as 227 generalized medical context rules from medspaCy. A complete set of the rules incorporated into the final model is available in the supplementary JSON files.

Ischemia has a variety of synonyms in cardiac stress test reports, and pertinent terminology may change the meaning of very similar phrases (Table 1). Of note, myocardial ischemia can occur in one myocardial territory without concomitant ischemia in another; a single cardiac stress test report can contain both negative and positive results for different myocardial territories. Any indication of ischemia in any territory is considered a binary positive for the purposes of clinical decision-making, even if other territories are negative. To account for this, positive for ischemia was weighted more highly than negative in subsequent token analyses. The rules-based NLP system produced a binary indicator for ischemia as well as a text excerpt of the targets and modifiers used in the determination. Supplemental Fig. 1 contains pseudocode for rules-based determination of ischemia.

**Table 1:** Example phrases that indicate or refute presence of ischemia

Example phrase	Ischemia label
Defect resolves on prone imaging, consistent with attenuation artifact	No
Fixed area of mildly reduced counts	No
Fixed perfusion defects are seen	No
Large perfusion defect without evidence of reversibility	No
Likely ischemia	Yes
Moderate area of reversibility	Yes
Moderate size, mildly reduced counts in anterior wall which is reversible	Yes
Negative for reversible ischemia	No
Negative myocardial perfusion study	No
Small perfusion abnormality is favored to be artifactual	No
Small, primarily fixed defect and a mild, reversible defect	Yes
There is a medium size, partially reversible defect	Yes
There is a small area of fixed ischemia	No

## Results

We identified 1,692,171 relevant stress test reports consisting of 1,096,341 unique patients (Table 2); a patient may have multiple stress tests with different results throughout their clinical journey. A practicing interventional cardiologist annotated a total sample of 654 stress test reports, each representing a unique patient. The training data consisted of 440 texts with a found 17% occurrence of ischemia by physician annotation. The second set consisted of 214 texts with a found 43% occurrence of ischemia by physician annotation. This second dataset was randomly broken up into validation and test sets, 115 and 99 texts respectively, for an overall 67% training, 18% validation, and 15% test data split. Demographic information for the training and validation/test sets is available in Supplemental Table 1.

**Table 2:** Demographics of veterans receiving cardiac stress tests in VA fiscal years 2011–2023

Demographic category	Frequency	Percentage
*Race/Ethnicity*		
White	755,246	68.9%
Black or African American	205,288	18.7%
Hispanic or Latino	62,888	5.7%
American Indian or Alaska Native	6719	0.6%
Native Hawaiian or Other Pacific Islander	6641	0.6%
Asian	6355	0.6%
Unknown	53,204	4.9%
*Sex*		
Male	1,024,523	93.4%
Female	71,641	6.5%
Unknown	177	0.02%
*Age at Time of First Stress Test in Years*		
18 to 40	26,509	2.4%
41 to 50	80,310	7.3%
51 to 60	231,971	21.2%
61 to 70	457,562	41.7%
71 to 80	232,428	21.2%
80 and Older	67,381	6.1%
Unknown	180	0.02%
Mean (SD)	64.5	(10.7)
Median	65.0	
*Total n*	1,096,341	100%

Additionally, though the BERT models truncate texts after 512 tokens, only 9 annotated texts (1.4%) exceeded 2048 characters, the average 512-token character count of 4 characters per token. As each LLM uses a slightly different tokenizer, this number is an appropriate approximation. The average number of characters in the physician-annotated impressions is 355 (std dev 446) with a median of 270.

The validation metrics against the test set across the various NLP systems are shown in Table 3. The LLM with the highest F1 score, ClinicalBERT, had a precision of 86.4%, recall of 100%, and F1 of 92.7% on the test dataset. The rules-based NLP was found to have an overall precision of 88.1%, recall of 97.4%, and F1 of 92.5% on the test dataset. These two approaches yielded similar performance in terms of F1, while the recall for the LLM approach was higher and the precision of the rules-based approach was higher. The results by VHA region are available in Supplemental Table 2, and the hyperparameters used can be found in Supplemental Table 3.

**Table 3:** Statistics for NLP model results compared to test data

Parameter	Formula	Rules-based model	Bio + Clinical BERT	Clinical BERT	DistilBERT Base Cased	DistilBERT Base Uncased
True Positives	a	37	33	38	30	35
False Positives	b	5	6	6	5	5
False Negatives	c	1	5	0	8	3
True Negatives	d	56	55	55	56	56
Total	a + b + c + d	99	99	99	99	99
Sensitivity/Recall	a/(a + c)*100%	97.4%	86.8%	100%	78.9%	92.1%
Specificity	d/(b + d)*100%	91.8%	90.2%	90.2%	91.8%	91.8%
Precision/Positive Predictive Value	a/(a + b)*100%	88.1%	84.6%	86.4%	85.7%	87.5%
Negative Predictive Value	d/(c + d)*100%	98.2%	91.7%	100%	87.5%	94.9%
F1	2*Precision*Recall/(Precision + Recall)	92.5%	85.7%	92.7%	82.2%	89.7%

The full source dataset was analyzed by the rules-based NLP system, which determined ischemia was present in 21.3% of the texts. The LLM was found to be too resource-intensive to run on the full dataset at the time.

## Discussion

In this study, we demonstrate two automated text processing methods for identifying ischemia status from cardiac stress test reports. Machine learning and rules-based approaches were validated in accordance with a physician-reviewed hold-out test dataset. Both methods achieved sufficient accuracy for clinical determination of the appropriateness of interventional cardiology methods of heart disease diagnosis and treatment, and the rules-based system is now in active operational use for patient healthcare quality and safety assessments.

Each approach has benefits and drawbacks. Due to the ability of transformer LLMs to utilize transfer learning, deep learning with fine-tuning alone can now succeed with far less data and computational capability than previously possible [23], in addition to the time and expertise saved on writing rules. However, when working with patient data, HIPAA regulations must be strictly adhered to, including data transmission and storage, precluding many available cloud-based platforms and requiring that any trained models stay within their training healthcare system. Furthermore, computational limits are still less likely to affect rules-based systems, a particularly relevant fact in the public sector. Moreover, decisions made by an LLM exist in a “black box” where the logic behind decision-making is veiled to the human users. Finally, LLMs have token limits, after which the texts must be broken up into pieces, losing context and possibly separating ideas that may be important [29].

Rules-based methods excel at analyzing highly structured texts and those with defined entities and context cues, rely on human knowledge, and require less computational power to scale up [30]. An easily interpretable system of rules written by a human can provide clarity when doctors and health care organizations make treatment and quality control decisions based on data provided by NLP. However, rules can become less relevant or obsolete as shifts in language occur over time, leading to poor performance without periodic language drift reviews [31]. After consideration of the advantages and disadvantages, we chose to operationalize the rules-based system.

Given that the LLM system showed higher recall and the rules-based approach showed higher precision, a hybrid approach may be warranted. Future work would benefit from evaluating different forms of hybrid systems to further optimize accuracy of ischemia identification. Additionally, more training data and computational power could be added to further fine-tune and run the LLMs, though annotation availability is limited by the time constraints of team practicing physicians.

Ongoing operational projects in VHA such as this one must consider the large and varied nature of the medical organization and its data. Using NLP to process these texts can be a challenging task: decades of data with consistently innovating medical procedures and terminologies, each medical center having its own lexicon and text structures, and millions of patients’ worth of information every year. This effort is worthwhile; readily available measures of ischemia for all sites through the utilization of NLP improve patient care and quality measures.

## Limitations

Our study has limitations. Only one annotator assessed the stress test report text data, preventing interrater agreement measures. The two datasets used were selected by different methods, and the training, validation, and test sets could have benefited from stratified sampling; this could not be accomplished due to the rules-based model development already occurring on the full training set. Our patient population of veterans in the United States is more likely to be male and older in age than America at large, potentially limiting the generalizability of this study. LLMs rapidly evolve, and as such these results may not reflect the newest and best-performing models available.

## Supplementary Information

Supplementary Material 1

Supplementary Material 2

Supplementary Material 3

Supplementary Material 4

Supplementary Material 5

## Data Availability

The datasets generated and/or analyzed during the current study are not publicly available due to HIPAA regulations that ensure the confidentiality of patient data.

## Abbreviations

BERT: Bidirectional encoder representations from transformers
HIPAA: Health insurance portability and accountability act
LLM: Large language model
NLP: Natural language processing
VHA: Veterans health administration
