# Supplementary material for: Utilizing large language models and natural language processing to classify ischemia status from cardiac stress tests in a large multicenter healthcare system
Source: BMC Res Notes. 2025 Dec 3;19:10. doi: 10.1186/s13104-025-07586-5 (PMC12781265; doi:10.1186/s13104-025-07586-5)
Supplement: Supplementary file 5 — Supplementary Material 5 [file 13104_2025_7586_MOESM5_ESM.docx]

Supplementary Material for “Utilizing large language models and natural language processing to classify ischemia status from cardiac stress tests in a large multicenter healthcare system”

Supplemental Table 1: Demographics of training and validation/test sampled data sets

| ***Dataset*** | ***Training Set*** | | ***Validation/Test Set*** | |
| --- | --- | --- | --- | --- |
| **Demographic Category** | **Frequency** | **Percent** | **Frequency** | **Percent** |
| *Race/Ethnicity* |  |  |  |  |
| White | 318 | 72.3% | 152 | 71.0% |
| Black or African American | 79 | 18.0% | 36 | 16.8% |
| Hispanic or Latino | 19 | 4.3% | 17 | 7.9% |
| Other | 7 | 1.6% | 5 | 2.3% |
| Unknown | 17 | 3.9% | 4 | 1.9% |
| *Sex* |  |  |  |  |
| Male | 413 | 93.9% | 205 | 95.8% |
| Female | 27 | 6.1% | 9 | 4.2% |
| *Age at Time of First Stress Test in Years* |  |  |  |  |
| 18 to 50 | 42 | 9.5% | 16 | 7.5% |
| 51 to 60 | 94 | 21.4% | 41 | 19.2% |
| 61 to 70 | 179 | 40.7% | 88 | 41.1% |
| 71 to 80 | 101 | 23.0% | 59 | 27.6% |
| 80 and Older | 23 | 5.2% | 10 | 4.7% |
| Unknown | 1 | 0.2% | 0 | 0.0% |
| Mean (SD) | 64.4 | 10.8 | 65.4 | 10.1 |
| Median | 65 |  | 66 |  |
| *Total n* | 440 | 100% | 214 | 100% |

Supplemental Table 2: Model results by VHA region

|  | ***Rules-Based Model Statistics by Region*** | | | | ***ClinicalBERT Model Statistics by Region*** | | | |
| --- | --- | --- | --- | --- | --- | --- | --- | --- |
| **Parameter** | **Pacific** | **Continental** | **Southeast** | **Northeast** | **Pacific** | **Continental** | **Southeast** | **Northeast** |
| True Positives | 6 | 12 | 14 | 5 | 6 | 12 | 14 | 6 |
| False Positives | 2 | 0 | 3 | 0 | 1 | 0 | 4 | 1 |
| False Negatives | 0 | 0 | 0 | 1 | 0 | 0 | 0 | 0 |
| True Negatives | 10 | 11 | 26 | 9 | 11 | 11 | 25 | 8 |
| Total | 18 | 23 | 43 | 15 | 18 | 23 | 43 | 15 |
| Sensitivity/ Recall | 100% | 100% | 100% | 83.3% | 100% | 100% | 100% | 100% |
| Specificity | 83.3% | 100% | 89.7% | 100% | 92% | 100% | 86% | 89% |
| Precision/ Positive Predictive Value | 75.0% | 100% | 82.4% | 100% | 86% | 100% | 78% | 86% |
| Negative Predictive Value | 100% | 100% | 100% | 90.0% | 100% | 100% | 100% | 100% |
| F1 | 85.7% | 100% | 90.3% | 90.9% | 92% | 100% | 88% | 92% |

Supplemental Table 3: Hyperparameters used for best fine-tuned LLMs

| **Hyperparameter** | **Bio+Clinical BERT** | **ClinicalBERT** | **DistilBERT Base Cased** | **DistilBERT Base Uncased** |
| --- | --- | --- | --- | --- |
| Learning Rate | 9.71E-05 | 5.27E-05 | 6.63E-05 | 9.49E-05 |
| Batch Size | 10 | 6 | 12 | 8 |
| Epochs | 3 | 4 | 4 | 4 |
| Weight Decay | 0.087 | 0.016 | 0.101 | 0.008 |
| Adam Beta 1 | 0.773 | 0.813 | 0.785 | 0.815 |
| Adam Beta 2 | 0.902 | 0.925 | 0.975 | 0.988 |
| Adam Epsilon | 1.04E-14 | 2.77E-09 | 5.03E-13 | 1.65E-13 |

Supplemental Figure 1: Pseudocode for rules-based determination of ischemia

For each document:

Tag all target and relevant context modifier words using rules

Sort target words into subject matter lists

If any target words have the “incomplete” category:

Return document negative

Go through the first subject list in order out of [ischemia, perfusion, defects/abnormalities, or test/study] with length > 0, create denominator variable equal to the list length and numerator variable equal to 0

For each target word in list:

For each modifier:

If modifier is negative:

Keep numerator and denominator the same

Elif modifier is positive:

Add one to numerator and denominator

Elif modifier is indeterminate:

Return document negative

If no modifiers are negative:

Add one to numerator, keep denominator the same

If numerator/denominator >= 50%, return positive for ischemia, else negative
